# Clinical characteristics and surgical approach for pituitary granular cell tumors: a case series of six patients and literature review

**DOI:** 10.3389/fonc.2025.1490783

**Published:** 2025-08-11

**Authors:** Jun Liu, Wenjun Zhang, Qianliang Huang, Xinyun Ye, Guanlin Huang

**Affiliations:** ^1^ Department of Neurosurgery, the 2nd Affiliated Hospital, Jiangxi Medical College, Nanchang University, Nanchang, Jiangxi, China; ^2^ Department of Neurosurgery, The Ganzhou Affiliated Hospital, Jiangxi Medical College, Nanchang University, Ganzhou, Jiangxi, China; ^3^ Department of Rehabilitation Medicine, The Ganzhou Affiliated Hospital, Jiangxi Medical College, Nanchang University, Ganzhou, Jiangxi, China

**Keywords:** granular cell tumors, neurohypophysis, imaging features, pathological diagnosis, prognosis

## Abstract

**Objective:**

To investigate the clinical characteristics and therapeutic approaches for granular cell tumors (GCT) of the neurohypophysis.

**Materials and methods:**

Retrospective case series and analyzed the clinical data of six patients with histopathologically confirmed GCT of the neurohypophysis, also conducting a simple review of relevant literature.

**Results:**

The median age at diagnosis for the cohort of six patients was 41.0 ± 11.73 years, with an age range of 21.8 to 52.7 years. A predominance of female patients was noted, accounting for five out of six cases. The most common clinical symptoms were headache and visual disturbances, each reported in five of the six patients. Magnetic resonance imaging (MRI) of the brain predominantly revealed a rounded morphology, and well-defined boundaries. Of these tumors, two were located in the suprasellar region while four were situated within the sellar region, encompassing intrasellar, suprasellar, and parasellar locations. Contrast-enhanced MRI demonstrated heterogeneous enhancement in four cases and homogeneous enhancement in two cases. Surgical intervention, either through a neuro-endoscopic endonasal transsphenoidal approach or craniotomy, achieved total or subtotal tumor resection in all patients. Postoperative histopathological examination confirmed the diagnosis of GCT in each instance. All patients participated in follow-up evaluations, during which varying degrees of clinical symptom improvement were documented. Importantly, none of the four patients who underwent complete tumor resection exhibited recurrence or metastasis.

**Conclusion:**

GCT of the neurohypophysis are rarely encountered clinical practice. The definitive diagnosis of GCT primarily relies on histopathological evaluation. Currently, the standard therapeutic approach involves complete surgical excision of the tumor using a neuroendoscopic endonasal transsphenoidal technique. Post-resection, the rates of recurrence and metastasis are significantly low.

## Introduction

Granular cell tumor (GCT) of the neurohypophysis is a rare type of pituitary cell tumor with a cytoplasm rich in eosinophilic granules and is considered an epithelial tumor of low malignant potential (1). The prevailing view is that the specialized neuroglial cells in the neurohypophysis serve as their source of origin (2). GCT of the neurohypophysis mainly occurs in young and middle-aged female patients, most patients with GCT of the neurohypophysis are found by medical examination when they present with visual field disorders and endocrine dysfunction (2, 3). The differentiation of GCT of the neurohypophysis from other sellar region tumors poses a clinical challenge due to overlapping imaging characteristics, it is clinically difficult to distinguish GCT in the pituitary gland from other sellar tumors (4). Currently, there is a lack of standardized clinical guidelines for the diagnosis and treatment, particularly in the domains of imaging diagnostics, histopathological assessments, therapeutic interventions, and prognostic evaluations. Therefore, it is essential to accumulate clinical data from a larger cohort of patients to thoroughly characterize the clinical characteristic features of GCT of the neurohypophysis.

In this retrospective case series, we conducted an analysis of clinical data from six patients diagnosed with GCT of the neurohypophysis, confirmed through histopathological examination, between January 2015 and January 2022. Additionally, we performed a review of pertinent literature. Our analysis concentrated on the clinical characteristics, imaging manifestations, pathological diagnoses, treatment strategies and prognostic outcomes of the patients.

## Materials and methods

### Participants and data collection

This retrospective study analyzed the clinical data of six patients who underwent surgical resection and were pathologically diagnosed with GCT of the neurohypophysis between January 2015 and January 2022. All six patients were diagnosed with GCT of the neurohypophysis following an independent review of the pathological sections conducted by two neuropathologists. To further substantiate the diagnosis, all archived pathological slides were reexamined and review meticulously reexamined by an experienced neuropathologist. The neuropathologist carefully assessed the slides, ensuring accuracy and precision, and referenced the to the World Health Organization (WHO) tissue classification guidelines to accurately identify the specific type of GCT present (5). This meticulous review and classification process ensure the reliability and validity of the diagnoses. The electronic medical record was retrieved based on the admission number, and comprehensive documentation of clinical information, surgical procedures, and imaging examinations for each patient was recorded. The image information was obtained from the picture archiving and communication system of the imaging center at our institution. All patients underwent preoperative cranial magnetic resonance imaging (MRI) scans to facilitate subsequent follow-up, with the radiological data being reassessed by two radiologists. Comprehensive clinical information, pertinent imaging data, treatment protocol, histological findings, and prognostic data of the patients were collected. The enrolled patients were followed up by telephone. According to the different stages of diseases, treatments, and complications, the content of follow-up visits varies. Follow-up assessments included evaluations of the patients’ general condition, laboratory tests (including pituitary associated hormones, and prolactin levels) and imaging studies such as brain MRI. Additionally, any instances of recurrence or metastasis were documented, noting the timing and location of recurrence as well as the current state of the disease. This study obtained approval from the hospital’s ethical review committee.

### Statistical method

Statistical analysis was performed using the SPSS26.0 statistical software (IBM Corp.). Data are expressed as the mean ± standard deviation, while count data are expressed in percentages.

## Results

### Patient characteristics and clinical findings

*The characteristics and clinical findings of the six patients are summarized in* **Table 1**. The median age at diagnosis was 41.0 ± 11.73 years (range: 21.8-52.7 years), with a predominance of female patients (83.3%). Among the patients, six presented with varying degrees of nonspecific headache symptoms, five exhibited visual disturbances, three experienced menstrual irregularities, and three reported polyuria. Notably, none of the patients experienced an acute onset or rapid progression of symptoms. The median duration of the disease at the time of consultation was 4.25 years (range: 0.3–10 years). Preoperative endocrine function tests were conducted on all six patients, including evaluations of pituitary hormones, plasma cortisol, plasma adrenocorticotropic hormone (ACTH), sex hormones, and thyroid function hormones, as detailed in **Table 2**. Preoperative laboratory tests indicated normal electrolyte levels in all patients. An increase in serum prolactin (PRL) was observed in three patients, while only one patient exhibited no discernible abnormalities in endocrine function.

**Table 1:** Summary of patient characteristics and clinical findings.

Patient	Gender	Age/year	Symptoms	Course of disease/year	24-hour urine volume	Previous medical history
1	F	43.5	Headache, diminution of vision	10	Normal	/
2	F	52.7	Headache	0.3	4800-5900ml/d	Treated with oral desmopressin
3	F	51.8	Headache, diplopia	2.6	Normal	/
4	F	21.8	Headache, diminution of vision, irregular menstruation	3.5	Normal	/
5	M	33.5	Headache, blurred vision	6	3100-3700ml/d	Treated with oral desmopressin
6	F	42.7	Headache, blurred vision, irregular menstruation	8	2800-3600ml/d	Treated with oral desmopressin

**Table 2:** The findings from the endocrine examinations conducted on six patients.

Hormones Follow-up time Patient		FSH	LH	PRL	GH	ACTH	FC	FT3	FT4	TSH
1	Preoperative	4.62	4.59	470	0.343	41.62	174.64	5.51	13.19	1.021
	Three-day postoperative	8.65	5.65	9.52	0.421	16.11	304.89	4.25	17.31	1.229
	One-month postoperative	5.73	5.97	10.01	0.388	30.01	200.83	4.97	10.01	1.96
	Three-month postoperative	4.41	6.81	13.11	0.237	19.14	250.56	4.54	11.24	1.71
2	Preoperative	1.09	0.35	6.87	0.317	8.36	440.85	3.87	10.4	0.227
	Three-day postoperative	2.26	0.53	4.59	0.283	12.48	421.37	3.8	10.4	0.195
	One-month postoperative	3.74	1.75	12.65	0.275	18.36	301.18	3.99	15.53	0.89
	Three-month postoperative	4.51	2.24	5.43	0.363	22.59	287.82	4.11	13.42	0.93
3	Preoperative	10.17	5.43	5.89	0.391	44.51	230.59	5.01	11.45	3.56
	Three-day postoperative	8.94	4.21	4.67	0.215	27.13	360.63	3.45	8.65	4.32
	One-month postoperative	9.79	8.12	8.84	0.191	38.68	400.14	4.67	10.41	3.71
	Three-month postoperative	11.21	6.77	7.08	0.213	40.13	357.71	5.12	12.16	3.15
4	Preoperative	12.08	4.69	50.18	0.231	37.21	402.81	3.65	13.97	1.331
	Three-day postoperative	9.54	4.24	8.49	0.152	19.72	408.95	3.23	24.3	0.273
	One-month postoperative	8.87	5.67	40.34	0.111	31.26	404.78	4.51	10.98	1.21
	Three-month postoperative	8.79	5.82	9.08	0.198	29.73	388.13	3.97	10.87	1.34
5	Preoperative	1.67	0.59	10.96	0.051	19.58	421.35	6.32	8.89	2.56
	Three-day postoperative	1.21	1.01	5.66	0.039	12.08	168.14	4.57	8.21	3.21
	One-month postoperative	2.98	2.34	9.23	0.041	23.21	245.93	5.11	8.34	3.66
	Three-month postoperative	2.34	3.51	8.54	0.051	29.04	345.12	5.19	8.66	3.49
6	Preoperative	4.98	1.13	72.67	0.061	7.16	473.16	4.99	10.12	4.31
	Three-day postoperative	4.77	1.78	6.32	0.058	19.08	114.35	5.49	11.23	3.87
	One-month postoperative	6.53	2.93	4.13	0.049	22.73	389.56	4.47	12.31	3.12
	Three-month postoperative	10.58	2.89	8.75	0.063	15.26	437.42	4.89	10.89	3.91
The normal reference values		**1.27-19.26 mIU/ml**	**1.24-8.62 mIU/ml**	**2.64-13.13 ng/ml**	**0.003-0.971 ng/ml**	**0-46 pg/ml**	**8Am 184.92-623.76 nmol/l**	**3.28-6.47 pmol/l**	**7.64-16.03 pmol/l**	**0.49-4.91 UIU/ml**

Bold values indicate the normal reference ranges for the corresponding laboratory parameters.

### Radiological findings

**Figure 1:**
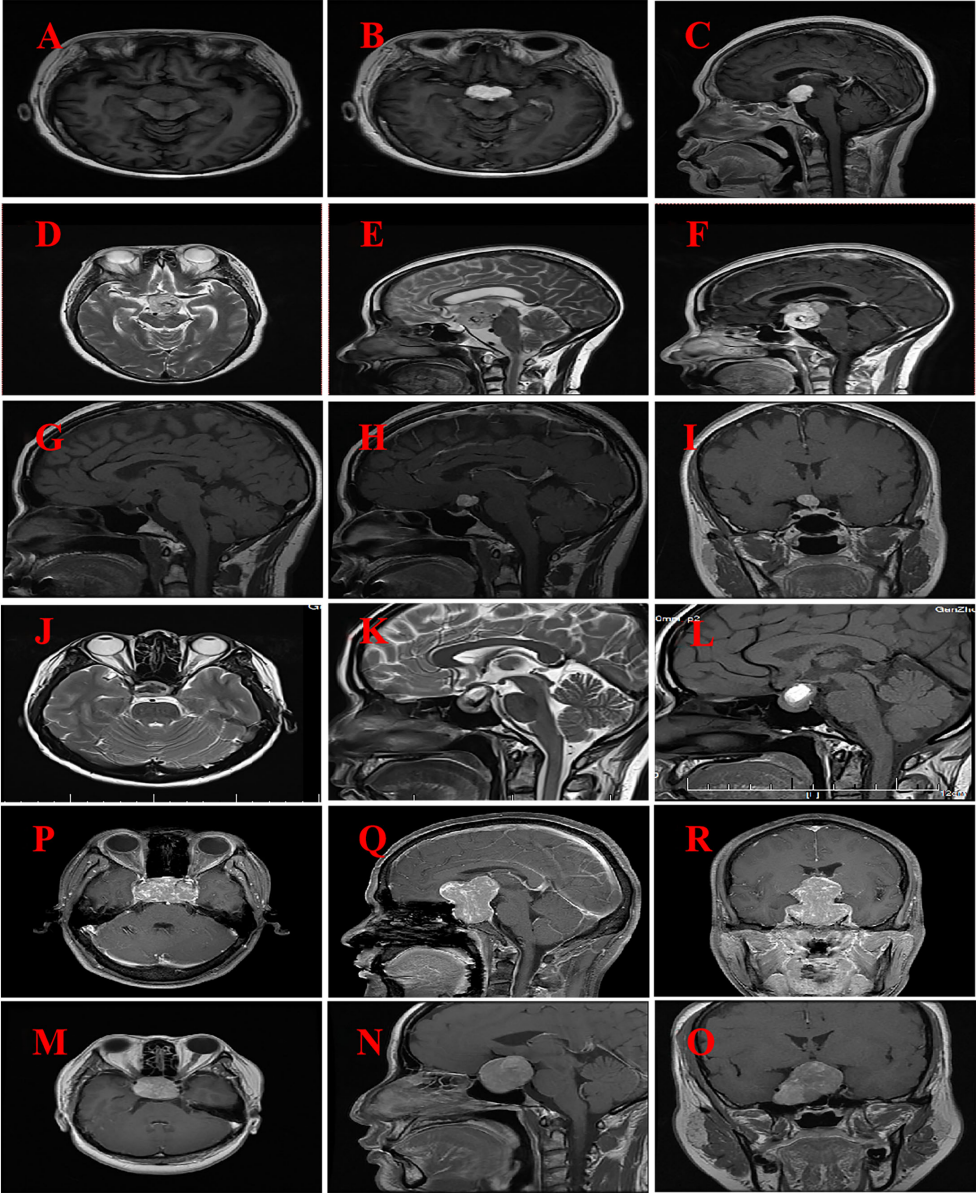
MRI imaging for six patients with GCT of the neurohypophysis. **(A–C)** The imaging reveals a well-defined, rounded lesion in the suprasellar region, characterized by a slightly prolonged T1 signal. Post-contrast enhancement demonstrates uniform and pronounced enhancement of the lesion, which appears to be in close proximity to the optic chiasm. (Case No. 1). **(D–F)** There are irregular lesions in the supratentorial region, tri-cerebral ventricles, and anterior pontine pools, characterized by slightly prolonged T2 signals and distinct borders. The enhancement scan reveals uneven and significant enhancement, closely associated with compression of the left thalamus and brainstem. (Case No. 2). **(G–I)** A rounded lesion located in the suprasellar region exhibited iso-intensity on T1-weighted imaging (T1WI), with well-defined margins. Post-contrast imaging demonstrated significant homogeneous enhancement of the lesion, resulting in compression and elevation of the optic chiasm. (Case No. 3). **(J–L)** Irregular lesions are observed in the sellar and suprasellar regions, characterized by short T2-weighted signal intensities and well-defined margins. Post-contrast imaging reveals heterogeneous enhancement of the lesions, with evidence of compression and elevation of the optic chiasm. (Case No. 4). **(M–O)** Solid lesions were observed in the saddle and suprasellar regions, exhibiting mild to moderate enhancement upon contrast-enhanced imaging. These lesions demonstrated bilateral invasion of the cavernous sinuses, encirclement of the internal carotid arteries, and upward extension into the suprasellar cisterns and third ventricles. Additionally, there was compression of the pituitary stalks and optic chiasm. (Case No. 5).**(P–R)** Solid lesions are observed both within and on the saddle. Upon enhanced imaging, these lesions exhibit moderate to significant enhancement, characterized by heterogeneous enhancement patterns. The lesions extend into the cavernous sinuses bilaterally, resulting in compression of the pituitary stalk and optic chiasm. (Case No. 6).

**Figure 2:**
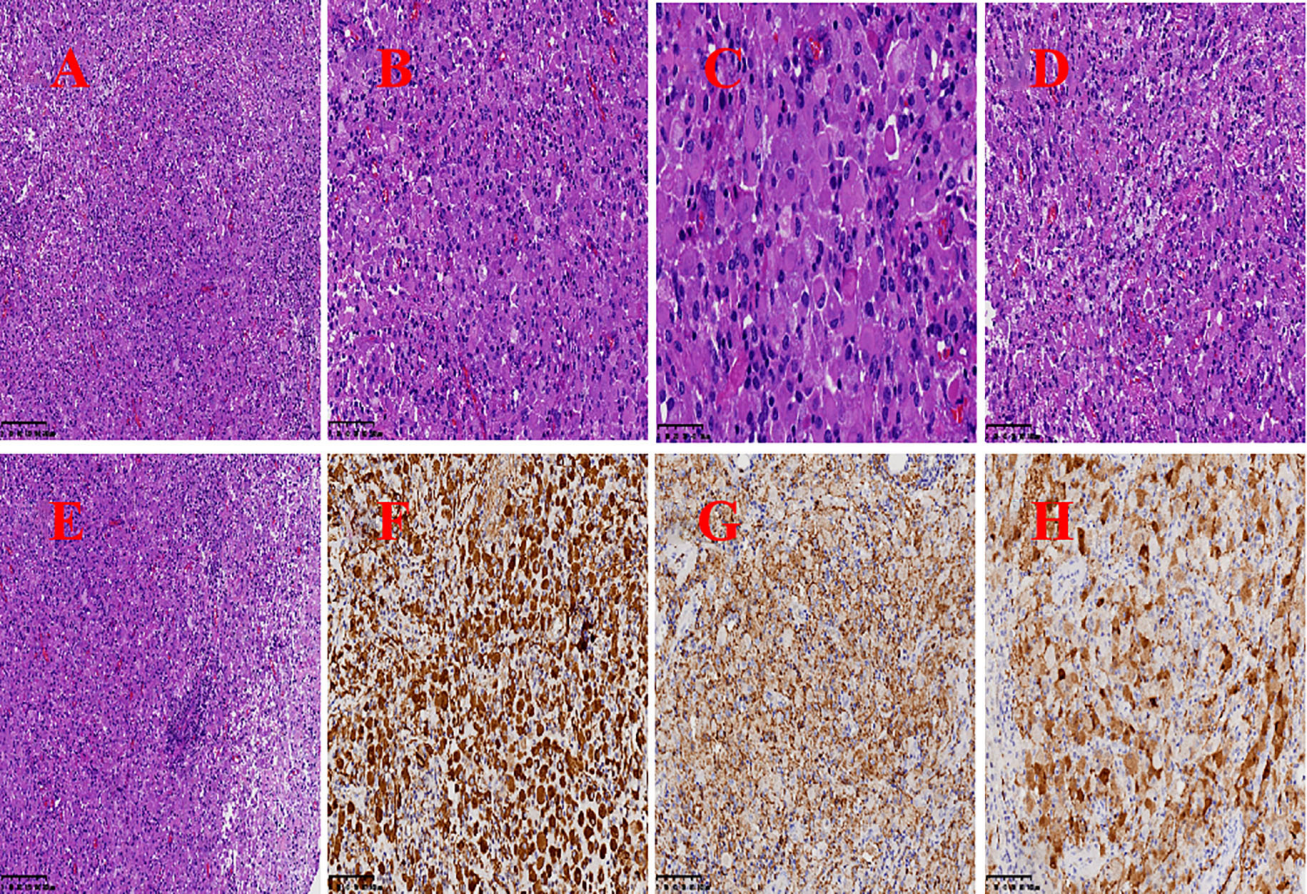
The clinicopathological features of GCT. **(A)** The tumor tissue is diffusely distributed in a sheet-like pattern (HE 10×10). **(B)** The tumor cells are relatively large and exhibit a round, oval, or polygonal shape, with abundant cytoplasm that is eosinophilic and granular in appearance (HE 20×10). **(C)** The tumor cells possess abundant, eosinophilic, granular cytoplasm, with nuclei that are round or oval in shape. The nuclear membrane is smooth, and prominent nucleoli are visible (HE 40×10). **(D)** Foam cells are observed within the tumor tissue (HE 20×10). **(E)** Focal lymphocytic aggregates are observed in the interstitial areas of the tumor cells and around the blood vessel.(HE 10×10). **(F–H)** Tumor cells expressed VIM, CD68 and S-100 respectively (Envision two-step method 20×10). HE, hematoxylin-eosin staining.

*The findings of brain MRI from six patients are presented in* **Table 3** *and* **Figure 1**. The MRI scan revealed that the tumor predominantly exhibited a spherical morphology with well-defined margins. Among the patients, two presented with suprasellar tumors (**Figures 1A–F**), while the remaining four patients had tumors situated in the sellar region, encompassing intrasellar, suprasellar, and parasellar components, including two instances of cavernous sinus invasion (**Figures 2A–F**). In all six patients, the tumors demonstrated iso-intensity on T1-weighted MRI (T1WI). In contrast, T2-weighted imaging (T2WI) showed more varied signal intensities: two cases exhibited slightly decreased signal intensity, two cases showed slightly increased signal intensity, one case presented with equivalent signal intensity, and one case displayed a mixed pattern of high and low signal intensities. On contrast-enhanced MRI, the tumors exhibited either homogeneous or heterogeneous enhancement, with two tumors demonstrating homogeneous enhancement. The dimensions of the tumors were 3.1 cm × 4.5 cm × 5.7 cm and 3.5 cm × 2.7 cm × 4.3 cm, respectively. One tumor displayed signs of calcification, measuring 2.3 cm × 2.7 cm × 3.3 cm. Preoperative imaging diagnosis indicated pituitary adenoma in three cases, craniopharyngioma in one case, and glioma in one case.

**Table 3:** MRI features for six patients with GCT of the neurohypophysis.

Patient	Location	Size/cm	MRI	Margin	MRI enhancement	Pre-operative diagnosis
1	Suprasellar region	2.0×2.7×2.2	T1 isointense, T2 slightly hypointense	Well-demarcated	Homogeneous density	Lymphoma
2	Sellar region, suprasellar region	2.3×2.7×3.3	T1 isointense, T2 slightly hypointense	Well-demarcated	Heterogeneous density	Glioma
3	Suprasellar region	1.2×1.2×1.0	T1 isointense, T2 slightly hyperintense	Well-demarcated	Homogeneous density	Craniopharyngioma
4	Sellar region, suprasellar region, parasella region	2.8×2.2×1.0	T1 isointense, T2 slightly hyperintense	Well-demarcated	Heterogeneous density	Pituitary adenoma
5	Sellar region, suprasellar region, parasella region	3.1×4.5×5.7	T1 isointense, T2 hypointense, T2 mixed with hyperintense spaces	Well-demarcated	Heterogeneous density	Pituitary adenoma
6	Sellar region, suprasellar region, parasella region	3.5×2.7×4.3	T1 isointense, T2 isointense	Well-demarcated	Heterogeneous density	Pituitary adenoma

### Analysis of treatment, pathology and outcomes

*The treatment and outcomes of six patients are presented in* **Table 4**. All patients through either a craniotomy or an endoscopic endonasal transsphenoidal approach. Intraoperatively, the tumor was observed to be closely associated with the superior pituitary stalk and exerting compression on the adjacent optic chiasm. The tumor exhibited a gray-white to tan coloration, a firm texture, and was characterized by a well-defined boundary, a capsule, and a rich vascular supply. Initial tumor removal was performed through piecewise intracapsular decompression, followed by the blunt and sharp dissection of the interface between the capsule and the arachnoid membrane, as well as the blood vessels and optic chiasm adjacent to the tumor surface. Complete tumor resection was achieved in four cases, while subtotal resection was accomplished in two cases. The primary limitation to achieving total resection in the latter cases was the tumor’s invasion into the cavernous sinus, coupled with the surgical techniques available at the time, which precluded total resection under microscopic guidance. Postoperative MRI confirmed complete resection in four cases and subtotal resection in two cases. None of the six patients received adjuvant radiotherapy or chemotherapy following surgery.

**Table 4:** Treatment and outcomes for six patients with GCT of the neurohypophysis.

Patient	Operative treatment	Post- operative complications	Results on follow-up
1	Radical excision	Transient diabetes insipidus, normal endocrine	No recurrence for 6 years
2	Radical excision	Hypopituitarism	No recurrence for 1 years
3	Radical excision	Favourable prognosis	No recurrence for 5 years
4	Radical excision	Transient diabetes insipidus	No recurrence for 3.5 years
5	Subtotal resection	Persistent diabetes insipidus, blurred vision, hypopituitarism	Recurrence 2 years after initial surgery, no recurrence 1.5 years after reoperation
6	Radical excision	Persistent diabetes insipidus, hypopituitarism	No recurrence for 2.5 years

*The pathology of six patients presented in* **Table 5** *and* **Figure 2**. The resected tumor tissue was subjected to histopathological examination and immunohistochemical (IHC) staining, leading to a postoperative histopathological diagnosis of GCT. Microscopically, the tumor cells were seen to be diffusely distributed in sheets or lobules (**Figure 2A**), and the tumor cells were large, round, ovoid or polygonal, with abundant cytoplasm, eosinophilic, and granular, which are the typical pathological and histological features of pituitary GCT (**Figure 2B**). The nuclei were round or round-like, the nuclear membrane is smooth, the nucleolus is visible, and the nuclear chromatin is slightly coarse (**Figure 2C**). Some nuclei exhibited deviation, with less prominent nucleoli and evenly distributed chromatin. Additionally, small focal foam cells (**Figure 2D**)and aggregation of lymphocytes was seen around blood vessels in the tumor tissue (**Figure 2E**). IHC staining showed tumors expressed Vimentin (**Figure 2F**), CD68 (**Figure 2G**) and S-100 (**Figure 2H**). The Ki-67 proliferative index ranged from approximately 1% to 10%.

**Table 5:** Immunohistochemical results of six patients with neuro-hypophyseal GCT.

Patient	CK	VIM	GFAP	S-100	Syn	P53	CD68	Lys	CD56	PAS	Ki-67
1	**−**	**+**	**−**	**+**	**−**	**+**	**+**	**+**	**−**	**+**	10%
2	**−**	**+**	**−**	**+**	**−**	**−**	**+**	**+**	**+**	**−**	5%
3	**−**	**+**	**+**	**+**	**−**	**−**	**+**	**−**	**−**	**+**	1%
4	**−**	**+**	**−**	**+**	**−**	**−**	**+**	**−**	**−**	**+**	3%
5	**−**	**+**	**+**	**+**	**−**	**−**	**+**	**−**	**+**	**−**	3%
6	**−**	**+**	**−**	**+**	**−**	**−**	**+**	**−**	**−**	**−**	1%

**Figure 3:**
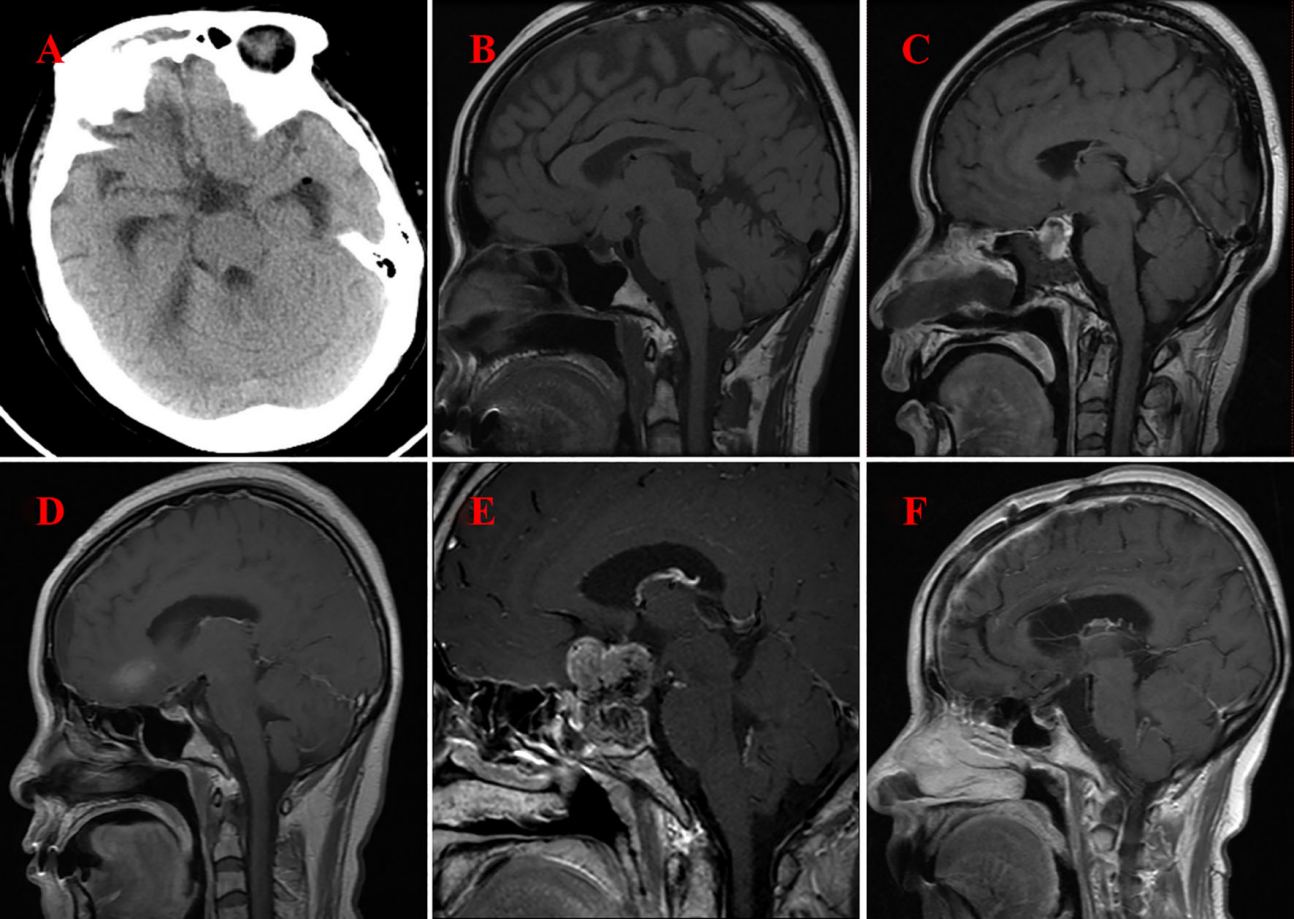
Imaging following surgery for GCT of the neurohypophysis. **(A)** CT scan of the brain indicates complete resection of the tumor located in the suprasellar region (Case No. 1). **(B)** MRI of the brain with contrast illustrates complete resection of the tumor within the prepontine cistern, suprasellar area, and the floor of the third ventricle (Case No. 2). **(C)** MRI of the brain with contrast reveals postoperative changes in the sellar region, characterized by minimal fluid accumulation (Case No. 3). **(D)** MRI of the brain with contrast again demonstrates complete resection of the tumor in the suprasellar region (Case No. 4). **(E)** MRI of the brain with contrast indicates the presence of residual and recurrent tumor in both the intrasellar and suprasellar regions(Case No. 5) **(F)** MRI of the brain with contrast confirms complete resection of the tumor in the intrasellar and suprasellar regions (Case No. 6).

The follow-up duration for all patients spanned from 1.5 to 6.0 years, with each individual successfully completing the period. **Figure 3** shows the follow-up imaging results of six patients. The follow-up assessments revealed that all six patients demonstrated varying degrees of improvement in preoperative symptoms, such as headaches and visual field disorders. One patient, who underwent subtotal resection, experienced a recurrence two years postoperatively and subsequently underwent a second total resection, with no recurrence observed during the ensuing 1.5-year follow-up. The remaining four patients who underwent total resection exhibited no signs of recurrence or metastasis throughout the follow-up period. Postoperatively, one patient developed transient diabetes insipidus, which resolved gradually during the follow-up period. In contrast, two patients developed persistent diabetes insipidus, and three patients exhibited hypopituitarism. These conditions were managed with the long-term administration of desmopressin acetate tablets and hormone replacement therapy.

## Discussion

Neuro-hypophyseal GCT is an uncommon epithelial neoplasm of the neurohypophysis, characterized by a potential for malignancy but generally demonstrating a relatively benign clinical course (1, 3, 6). As a distinct subtype of pituitary cell tumor, neuro-hypophyseal GCT is one of the three subtypes included in the World Health Organization’s pituitary tumor classification of 2022, with an incidence rate of approximately 0.5% among all tumors in the sellar region (5). Previous studies have indicated that neuro-hypophyseal GCT is most prevalent in individuals aged 40 to 50 years, is uncommon in pediatric patients, and rare during transitional age, with the youngest reported patient being 8 years old (7, 8). These tumors exhibit a higher prevalence in female patients, with a male-to-female ratio of approximately 1:2. In this study, six cases of neuro-hypophyseal GCTs represented 0.47% (6 out of 1,276) of all sellar tumors diagnosed in our department during the study period. The ages of the patients ranged from 21.8 to 52.7 years, with a median age of 42.1 years, and a male-to-female ratio of 1:5. While the age of onset is consistent with existing literature, further research with larger sample sizes is required to validate the observed gender disparity. The clinical manifestation of neuro-hypophyseal GCTs closely resemble those of other space-occupying lesions within the sellar region. These manifestations typically include headaches due to lesion compression, visual field disturbances, and symptoms associated with peritumoral tissue destruction and endocrine dysfunction (2, 3). Among the endocrine dysfunctions, hyperprolactinemia is the most prevalent symptom, often resulting from pituitary stalk compression and subsequent inhibition of dopamine secretion (9). In rare cases, patients may experience intra-tumoral hemorrhage, optic nerve edema, and hypoglycemic episodes (10–12). Owing to the indolent growth pattern of neuro-hypophyseal GCTs, many patients remain asymptomatic or experience only occasional nonspecific headaches during the early stages. By the time clinical symptoms become evident, the tumor is generally of substantial size (13). In the cohort under study, the clinical manifestations were as follows: (1) Changes in visual acuity and visual field were the most prominent symptoms. Specifically, five patients in this study presented with visual acuity and visual field disorders, which were the primary reasons for their hospital admission. (2) During the progression of the disease, headache emerged as a prevalent symptom. An analysis of the medical histories of the six patients indicated varying degrees of headache; however, ophthalmologic examinations conducted at the hospital did not identify any related abnormalities. (3) In this study, five patients exhibited abnormalities in endocrine function, with three cases each of hyperprolactinemia and polyuria symptoms. The GCT of the neurohypophysis gland demonstrates a lack of secretory function, and the abnormal endocrine function may be attributed to lesions originating from the pituitary stalk. Notably, one patient in this cohort has not yet exhibited additional symptoms indicative of pituitary dysfunction. It is hypothesized that, although the lesions originate from the pituitary stalk, the growth of the GCT has not exerted compression on the normal pituitary gland and pituitary stalk within a short timeframe. This is likely due to its location and development towards the sellar region, thereby mitigating potential endocrine dysfunction. (4) Postoperative recurrence of the tumor may occur, potentially due to its relationship with surrounding tissues, particularly when the tumor is closely adhered to the pituitary stalk or cavernous sinus, making complete surgical removal challenging. In the cases examined, the tumor had invaded the cavernous sinus, and despite the application of appropriate surgical techniques, complete resection was not feasible under microscopic guidance, leading to tumor recurrence observed during follow-up. Furthermore, given the tumor’s location in the intrasellar or suprasellar region, it can affect the pituitary gland and optic nerve. Its clinical manifestations, including symptoms, signs, and endocrine hormone test results, are often non-specific. Additionally, differentiating this tumor from other sellar region tumors using cranial MRI is difficult. In this study, discrepancies were observed between the preoperative and postoperative pathological diagnoses in all six cases, highlighting the challenges associated with the preoperative diagnosis of GCT of the neurohypophysis. The inconsistency between the preoperative and postoperative diagnoses suggests the inherent difficulty in accurately diagnosing GCT of the neurohypophysis prior to surgery (6) Larger tumor volumes tend to occupy the sellar turcica more extensively, potentially filling it completely, which increases the risk of misdiagnosing GCT of the neurohypophysis as a pituitary adenoma originating in the sellar turcica. (7) Histopathological examination remains the “gold standard” for the definitive diagnosis of GCT of the neurohypophysis. In this study, all six patients underwent surgical procedures to obtain tumor specimens, which were subsequently subjected to histopathological analysis to confirm the diagnosis.

A preoperative diagnosis of GCT of the neurohypophysis can be effectively established based on imaging findings, as these tumors exhibit distinct imaging characteristics. Typically, GCTs are located within the intrasellar and suprasellar regions, with occasional extension into the cavernous and sphenoid sinuses (14). On brain CT, GCTs of the neurohypophysis present as round and partially solid lesions, with relatively well-defined boundaries. These lesions appear slightly hyperdense compared to normal brain parenchyma, and demonstrate uniform enhancement following contrast administration (11). Notably, signs of necrosis, cystic changes, calcification, and hemorrhage are infrequently observed (15). MRI commonly reveals intrasellar and suprasellar space-occupying lesions, associated with the pituitary stalk, which may be linked to the origin of neuro-hypophyseal GCTs from specialized glial cells (16). On T1WI, most lesions exhibit iso-intensity, while on T2WI, they display iso-intensity or hypo-intensity. Larger lesions tend to show heterogeneous signal intensity (16, 17). The granular hypointense signal observed in the lesion on T2WI may be attributed to abundance of eosinophilic granules within the cytoplasm of the tumor cells, which aids in differential diagnosis (18). The lesions demonstrated either homogeneous or heterogeneous enhancement following contrast administration (15–17). In this cohort, the tumors of six patients exhibited iso-intensity on T1WI. On T2WI, two cases showed slightly decreased signal intensity, two cases showed slightly increased signal intensity, one case displayed iso-intensity, and one case exhibited a mixed pattern of high and low signal intensities. Across these six cases, the tumors consistently exhibited pronounced homogeneous or heterogeneous enhancement on post-contrast MRI scans. Furthermore, two cases presented with suspected intra-tumoral cystic degeneration, while one case demonstrated intra-tumoral hemorrhage. The MRI characteristics of GCT of the neurohypophysis in this cohort align with those previously reported in the literature. Differentiating GCT of the neurohypophysis from other lesions in the sellar region based solely on imaging modalities continues to pose a significant challenge. Furthermore, there is a lack of research on the application of advanced MRI techniques specifically for GCT of the neurohypophysis.

Due to the low incidence of GCT of the neurohypophysis, their nonspecific clinical presentation, and the heterogeneity of sellar region tumors, achieving a precise preoperative diagnosis of this rare condition remains challenging, despite thorough imaging assessments, evaluations of endocrine function, and tumor marker analyses. Previous literature has indicated that pituitary adenomas are most commonly diagnosed preoperatively. However, it is noteworthy that the occurrence of diabetes insipidus (DI) in case of pituitary adenomas is relatively uncommon (19). In this study cohort, three patients exhibited polyuria, which can be primarily attributed to the typical origin of GCTs in the posterior pituitary. These tumors are frequently located in the neurohypophysis, accounting for the higher incidence of DI among GCT patients. Furthermore, DI may precede the onset of other clinical symptoms, even in instances where the tumor is not detectable via neuroimaging. In patients presenting with symptoms of DI, certain imaging features may be discernible. Notably, the enlargement and enhancement of the pituitary stalk emerge as the most prevalent findings on MRI, in addition to the loss of the high signal intensity of the posterior pituitary on T1WI (20). Beyond the distinctive imaging features and clinical symptoms, early recognition of DI in such lesions is crucial, as it guides the diagnostic process towards identifying a non-adenomatous sellar/parasellar tumor and facilitates the prompt initiation of appropriate treatment (20). The definitive diagnosis of this condition, as well as the classification of cases within this cohort, was established through postoperative histopathological examination and IHC staining results. Microscopically, the tumor cells exhibited a polygonal, well-differentiated morphology, organized in nodular and sheet-like patterns, characterized by relatively small nuclei, inconspicuous nucleoli, and cytoplasm containing varying amounts of eosinophilic granules (1, 5). In addition to the forementioned histological characteristics, the presence of small focal foam cells and perivascular lymphocyte sleeve infiltration can be observed in the tissue (21). The IHC expression of GCTs in the neurohypophysis is variable. Most studies have demonstrated that tumor cells express S-100, CD68, and Vimentin, whereas markers such as CK, Syn, CgA, Desmin, and pituitary hormones are typical negative, with the majority of cases also showing negativity for GFAP (5, 21). In our cohort of six cases, all exhibited positive expression for S-100, CD68, and Vimentin. Additionally, there three cases showed positive PAS staining, Two cases were positive for GFAP and CD56, and one case demonstrated positive expression for P53. Negative expression was observed for CK, Syn, and pituitary hormone markers. Despite the inconsistencies in the expression of various IHC markers in GCTs of the neurohypophysis, IHC staining remains a valuable tool for distinguishing GCTs of the neurohypophysis from pituitary adenomas and craniopharyngiomas. Furthermore, the literature that thyroid transcription TTF-1 positivity is a significant feature of the immunophenotype of GCTs in the neurohypophysis (5). Regrettably, we were unable to detect TTF-1. Nonetheless, existing research indicates that TTF-1 lacks specificity, as it can also be positively expressed in other morphologically similar tumors such as spindle cell oncocytoma and sellar ependymoma. Consequently, this marker is of limited utility for the definitive diagnosis of GCTs of the neurohypophysis. Recent studies have demonstrated that TFE-3 is diffusely expressed in this tumor type (22). Chamberlain et al. (23) propose that the TFE-3 protein plays a role in regulating the emergency response of lysosomes/phagosomes and Golgi bodies. This expression may be attributed to the abundance of eosinophilic granules in the cytoplasm of tumor cells in neuro-hypophyseal GCTs. The histopathological features of tumor cells, characterized by prominent granular and eosinophilic cytoplasm, aid in distinguishing GCTs of the neurohypophysis from other TTF-1-positive tumors. When tumor cells in neuro-hypophyseal GCTs exhibit nuclear atypia and increased mitotic figures, they express PCNA, Ki-67, and p53, which are histopathological markers of anaplasia, suggesting a potential for malignancy (24). In the absence of definitive histomorphological or other prognostic indicators, such patients necessitate long-term follow-up and aggressive treatment strategies. The cellular morphology, immunohistochemical findings, and clinical presentation of the six cases discussed in this article align consistent with the diagnosis of benign GCT of the neurohypophysis. Among these cases, five patients exhibited positive Ki-67 indices ranging from 1% to 5%, while one patient demonstrated a higher Ki-67 positive at 10%, and positive P53 expression, indicating active tumor proliferation. Notably, no tumor recurrence has been observed following complete tumor resection and subsequent follow-up to date.

GCT of the neurohypophysis are classified as low-grade non-neuroendocrine tumors, with surgical resection being the treatment of choice (21). There is currently no consensus regarding the necessity of postoperative radiotherapy and chemotherapy, and the effectiveness of adjuvant therapies remains uncertain. A meta-analysis has demonstrated that for craniopharyngiomas located in the sellar region, endonasal transsphenoidal surgery utilizing neuro-endoscopy achieves resection and recurrence rates comparable to those of open craniotomy, while significantly reducing mortality rates (25). Endoscopic trans-nasal transsphenoidal surgery is characterized by minimal invasiveness, excellent visualization, and the capacity for close observation of the pituitary stalk and hypothalamus, thereby establishing it as the preferred and optimal surgical approach for GCT of the neurohypophysis. Nevertheless, it is imperative to avoid intraoperative iatrogenic injury to the pituitary gland, hypothalamus, optic nerve, internal carotid artery, and its branches. Surgical intervention can partially alleviate the compression symptoms exerted by the tumor on the pituitary stalk and optic chiasm, while also facilitating a definitive histopathological diagnosis to inform subsequent therapeutic strategies, thereby playing a pivotal role in achieving favorable prognostic outcomes. In this study, all six cases underwent endoscopic trans-nasal transsphenoidal surgery for tumor excision. The presence of arachnoid membranes in the middle and upper segments of the pituitary stalk, along with encapsulated nature of the tumor, resulted in minimal adhesion and the presence of an arachnoid interface during the procedure. These conditions were conducive to complete tumor resection and the preservation of the pituitary stalk and neurovascular structures. Consequently, postoperative pituitary function was preserved, surgery, and clinical symptoms improved to varying extents, yielding satisfactory therapeutic outcomes. In cases where visual impairment is attributed to tumor compression, most patients experienced improved vision postoperatively, consistent with outcomes reported in the literature (26). This suggests that endoscopic trans-nasal transsphenoidal surgery for GCT of the neurohypophysis is a relatively safe and effective treatment modality. However, when the tumor invades adjacent neurovascular structures or extends into the para-sellar and suprasellar regions, craniotomy may be required (13). In this cohort, one case involved a large tumor with a substantial blood supply and invasion of the bilateral cavernous sinuses, which precluded complete resection during surgery and necessitated the termination of the procedure. Postoperative MRI indicated near-total resection of the tumor. Subsequent follow-up visits revealed tumor recurrence, which was successfully managed with a second surgical intervention. This success was attributed to advancements in surgical techniques and equipment, as well as improvements in hemostatic and repair materials.

In previous studies, the majority of patients with GCT of the neurohypophysis did not exhibit metastasis, with only minority demonstrating recurrence and invasive growth. This suggests that GCT of the neurohypophysis is typical an indolent tumor with a favorable prognosis. However, research has indicated that certain GCTs extending beyond the cranial cavity may experience local recurrence post-surgery, as well as invasive growth and metastasis (27). Jiang et al. (12) reported that residual tumors following GCT surgery may recur. The effectiveness of adjuvant radiotherapy and chemotherapy following surgical intervention remains a topic of debate. A recent meta-analysis revealed no significant difference in recurrence rates between patients who received adjuvant radiotherapy and chemotherapy and those who did not (28). In the present cohort of six patients, none underwent radiation or chemotherapy following endoscopic tumor resection. Of these, five patients who achieved complete tumor resection have shown no signs of recurrence or metastasis on imaging to date, consistent with previous findings. Nonetheless, ongoing follow-up is necessary to assess the potential for further development or malignant transformation. The precise recurrence rate following surgical intervention remains undetermined. A review of the literature indicates that the majority of cases did not experience recurrence postoperatively, while only a minority of patients reported instances of recurrence. A significant contributing factor to recurrence is the incomplete resection of the tumor; additionally, some cases lacked follow-up data or resulted in mortality due to hypopituitarism and other comorbidities (13, 14, 26). Consequently, the absence of reliable data in prior studies precludes an accurate estimation of the surgical recurrence rate. Currently, research on GCT of the neurohypophysis is predominantly derived from case reports, which are notably limited in number. Although some individual cases have demonstrated favorable therapeutic outcomes, the clinical diagnostic and treatment protocols warrant further investigation.

This study is subject to several limitations. Firstly, the sample size is limited, attributable to the low clinical incidence rate of GCT in the neurohypophysis. Secondly, none of the patients in this study underwent TTF-1 or TFE-3 testing, despite the potential of these markers to enhance diagnostic accuracy for GCT in the neurohypophysis. Thirdly, the study lacks detailed information on surgical recurrence, potentially due to factors such as the small sample size, limited follow-up duration for certain cases, and the low incidence of malignant neuro-hypophyseal tumors. We hypothesize that the primary reason for this omission is the low malignant potential of GCTs in the neurohypophysis. Notably, two cases in this study with follow-up periods exceeding five years exhibited no recurrence, reinforcing the classification of neuro-hypophyseal GCT as is a low-grade malignant tumor. Consequently, we can inter that the recurrence rate of neuro-hypophyseal GCT is low, and surgical intervention is highly effective. Supporting this, a study analyzing clinical data from 141 patients with neuro-hypophyseal GCT reported an overall survival (OS) rate of 84.7% at five years (13). Patients presenting with tumors less than 2.5 cm in diameter demonstrated a superior five-year OS rate compared to those with tumors measuring 2.5 cm or greater. Furthermore, individuals who underwent complete tumor resection exhibited a higher five-year OS than those who received partial resection, were not subjected to surgical intervention, or only underwent tumor biopsy. The five-year progression-free survival rate was observed to be 80.8%. Notably, the administration of adjuvant radiotherapy did not result in a statistically significant enhancement of the OS of among the patient cohort.

In conclusion, GCT of the neurohypophysis represents a rare neoplasm that is not invariably indolent, predominantly affecting young and middle-aged women. The nonspecific nature of its clinical presentation and imaging characteristics poses significant challenges in distinguishing GCT of the neurohypophysis from other pituitary tumors preoperatively. Furthermore, its rarity contributes to a high risk of misdiagnosis, underscoring the necessity for further research to comprehensively delineate the attributes of neuro-hypophyseal GCT. While certain imaging features may aid in preoperative diagnostic predictions, definitive diagnosis remains reliant on histopathological and immunohistochemical evaluation. The prognosis for this tumor is generally favorable, with low rates of recurrence and metastasis following complete surgical resection. It is imperative for clinicians to enhance their understanding of this tumor to prevent misdiagnosis and appropriate management. In clinical practice, the possibility of neuro-hypophyseal GCT should be considered in all cases presenting with sellar or suprasellar masses.

## Data Availability

The original contributions presented in the study are included in the article/supplementary material. Further inquiries can be directed to the corresponding authors.

## References

[1] Khurana U Shrivastava A Jain R Goel G Joshi D Kapoor N. Squash smear cytology of pituitary granular cell tumor: A case report and review of literature with special emphasis on cytological differential diagnosis in pituitary region. Diagn Cytopathol. (2021) 49:E119–e124. doi: 10.1002/dc.24612, PMID: 32926559

[2] Han F Gao L Wang Y Jin Y Lv Y Yao Z. Clinical and imaging features of granular cell tumor of the neurohypophysis: A retrospective analysis. Med (Baltimore). (2018) 97:e9745. doi: 10.1097/MD.0000000000009745, PMID: 29489677 PMC5851750

[3] Cohen-Gadol AA Pichelmann MA Link MJ Scheithauer BW Krecke KN Young WF Jr.. Granular cell tumor of the sellar and suprasellar region: clinicopathologic study of 11 cases and literature review. Mayo Clin Proc. (2003) 78:567–73. doi: 10.4065/78.5.567, PMID: 12744543

[4] Thakkar K Ramteke-Jadhav S Kasaliwal R Memon SS Patil V Thadani P. Epari S et al: Sellar surprises: a single-centre experience of unusual sellar masses. Endocr Connect. (2020) 9:111–21. doi: 10.1530/EC-19-0497, PMID: 31910151 PMC6993267

[5] Asa SL Mete O Perry A Osamura RY. Overview of the 2022 WHO classification of pituitary tumors. Endocr Pathol. (2022) 33:6–26. doi: 10.1007/s12022-022-09703-7, PMID: 35291028

[6] Mohan A Kannoth P Unni C Jose BV Parambil RM Nandeesh BN. Rare neurohypophyseal tumor presenting as giant pituitary macroadenoma with cavernous sinus invasion - A case report and review of literature. Surg Neurol Int. (2020) 11:261. doi: 10.25259/SNI_316_2020, PMID: 33024599 PMC7533092

[7] Benites Filho PR Sakamoto D Machuca TN Serapião MJ Ditzel L Bleggi Torres LF. Granular cell tumor of the neurohypophysis: report of a case with unusual age presentation. Virchows Arch. (2005) 447:649–52. doi: 10.1007/s00428-005-1229-z, PMID: 16133355

[8] Feola T Pirchio RS Puliani G Pofi R Crocco M Sada V. Appetecchia M et al: Sellar and parasellar lesions in the transition age: a retrospective Italian multi-centre study. J Endocrinol Invest. (2023) 46:181–8. doi: 10.1007/s40618-022-01900-9, PMID: 36001286 PMC9829590

[9] Piccirilli M Maiola V Salvati M D’Elia A Di Paolo A Campagna D. Granular cell tumor of the neurohypophysis: a single-institution experience. Tumori. (2014) 100:160e–4e. doi: 10.1177/1636.17940, PMID: 25296610

[10] Maenhoudt W Van Dorpe J Vanhauwaert D. Granular cell tumor of the pituitary presenting with major intraventricular hemorrhage. World Neurosurg. (2020) 140:60–2. doi: 10.1016/j.wneu.2020.05.027, PMID: 32407921

[11] Pendharkar AV Lin CY Born DE Hoffman AR Dodd RL. Granular cell pituitary tumor in a patient with multiple endocrine neoplasia-1. Cureus. (2019) 11:e4541. doi: 10.7759/cureus.4541, PMID: 31275768 PMC6592835

[12] Jiang B Shi X Fan C. Sellar and suprasellar granular cell tumor of the neurohypophysis: A rare case report and review of the literature. Neuropathology. (2018) 38:293–9. doi: 10.1111/neup.12448, PMID: 29271018

[13] Ahmed AK Dawood HY Penn DL Smith TR. Extent of surgical resection and tumor size predicts prognosis ingranular cell tumor of the sellar region. Acta Neurochir(Wien). (2017) 159(11):2209–16. doi: 10.1007/s00701-017-3337-3, PMID: 28948361

[14] Guerrero-Pérez F Marengo AP Vidal N Iglesias P Villabona C. Primary tumors of the posterior pituitary: A systematic review. Rev Endocr Metab Disord. (2019) 20(2):219–38. doi: 10.1007/s11154-019-09484-1, PMID: 30864049

[15] Zhang Y Teng Y Zhu H Lu L Deng K Pan H. Granular cell tumor of the neurohypophysis: 3 cases and a systematic literature review of 98 cases. World Neurosurg. (2018) 118:e621–30. doi: 10.1016/j.wneu.2018.07.004, PMID: 30017767

[16] Sassi F Zehani A Slimane A Said IB Bellil K Haouet S. Supra-sellar granular cell tumor: Report of a case with literature review. Int J Surg Case Rep. (2023) 112:108977. doi: 10.1016/j.ijscr.2023.108977, PMID: 37883878 PMC10667900

[17] Shizukuishi T Abe O Haradome H Fukushima T Katayama Y Sugitani M. Granular cell tumor of the neurohypophysis with optic tract edema. Jpn J Radiol. (2014) 32:179–82. doi: 10.1007/s11604-013-0279-4, PMID: 24414885

[18] Liu HL Huang BY Zhang MS Wang HR Qu YM Yu CJ. Sellar and suprasellar granular cell tumor of neurohypophysis. Chin Med J (Engl). (2017) 130:741–3. doi: 10.4103/0366-6999.201605, PMID: 28303860 PMC5358427

[19] Harary M DiRisio AC Dawood HY Kim J Lamba N Cho CH. Endocrine function and gland volume after endoscopic transsphenoidal surgery for nonfunctional pituitary macroadenomas. J Neurosurg. (2019) 131:1142–51. doi: 10.3171/2018.5.JNS181054, PMID: 30497144

[20] Angelousi A Mytareli C Xekouki P Kassi E Barkas K Grossman A. Diabetes insipidus secondary to sellar/parasellar lesions. J Neuroendocrinol. (2021) 33:e12954. doi: 10.1111/jne.12954, PMID: 33769630

[21] Whipple SG Savardekar AR Rao S Mahadevan A Guthikonda B Kosty JA. Primary tumors of the posterior pituitary gland: A systematic review of the literature in light of the new 2017 world health organization classification of pituitary tumors. World Neurosurg. (2021) 145:148–58. doi: 10.1016/j.wneu.2020.09.023, PMID: 32916355

[22] Yang GZ Li J. Granular cell tumor of the neurohypophysis with TFE-3 expression: A rare case report. Int J Surg Pathol. (2017) 25:751–4. doi: 10.1177/1066896917712861, PMID: 28612665

[23] Chamberlain BK McClain CM Gonzalez RS Coffin CM Cates JM. Alveolar soft part sarcoma and granular cell tumor: an immunohistochemical comparison study. Hum Pathol. (2014) 45:1039–44. doi: 10.1016/j.humpath.2013.12.021, PMID: 24746209

[24] Singh VA Gunasagaran J Pailoor J. Granular cell tumour: Malignant or benign? Singapore Med J. (2015) 56:513–7. doi: 10.11622/smedj.2015136, PMID: 26451054 PMC4582131

[25] Qiao N. Endocrine outcomes of endoscopic versus transcranial resection of craniopharyngiomas: A system review and meta-analysis. Clin Neurol Neurosurg. (2018) 169:107–15. doi: 10.1016/j.clineuro.2018.04.009, PMID: 29655011

[26] Cole TS Potla S Sarris CE Przybylowski CJ Baranoski JF Mooney MA. Rare thyroid transcription factor 1-positive tumors of the sellar region: barrow neurological institute retrospective case series. World Neurosurg. (2019) 129:e294–302. doi: 10.1016/j.wneu.2019.05.132, PMID: 31132506

[27] Mohapatra A Potretzke AM Knight BA Han M Figenshau RS. Metastatic granulosa cell tumor of the testis: clinical presentation and management. Case Rep Urol. (2016) 2016:9016728. doi: 10.1155/2016/9016728, PMID: 27293952 PMC4884594

[28] Rubino F Martinez-Perez R Vieira S Voscoboinik DS Mural M Orr AJ. Granular cell tumors of the sellar region: what should be done after subtotal resection? A systematic review. Pituitary. (2020) 23:721–32. doi: 10.1007/s11102-020-01068-6, PMID: 32740679

